# An autopsy case of disseminated carcinomatosis of the bone marrow from esophageal adenocarcinoma

**DOI:** 10.1002/ccr3.7877

**Published:** 2023-09-20

**Authors:** Daisuke Suzuki, Shiori Meguro, Shin Furusawa, Nanako Hashimoto, Hideya Kawasaki, Isao Kosugi, Yasunori Enomoto, Mayu Fujihiro, Hiroe Tsukui, Toshihide Iwashita

**Affiliations:** ^1^ Department of Diagnostic Pathology Chutoen General Medical Center Shizuoka Japan; ^2^ Department of Regenerative and Infectious Pathology Hamamatsu University School of Medicine Shizuoka Japan; ^3^ Department of Nephrology Chutoen General Medical Center Shizuoka Japan; ^4^ Department of Radiology Chutoen General Medical Center Shizuoka Japan; ^5^ Institute for NanoSuit Research, Preeminent Medical Photonics Education & Research Center Hamamatsu University School of Medicine Shizuoka Japan; ^6^ Department of Diagnostic Pathology Hamamatsu University School of Medicine Shizuoka Japan

**Keywords:** autopsy, disseminated carcinomatosis of the bone marrow, disseminated intravascular coagulation syndrome, esophageal adenocarcinoma

## Abstract

**Key Clinical Message:**

Disseminated carcinomatosis of the bone marrow is rare. We present such a case, which is useful for raising awareness about the importance of early diagnosis and treatment of carcinomas complicated by disseminated carcinomatosis of the bone marrow.

**Abstract:**

This is the first autopsy report of disseminated carcinomatosis of the bone marrow (DCBM) in esophageal adenocarcinoma. Advanced poorly differentiated adenocarcinoma with signet ring cell carcinoma arising in Barrett's esophagus caused disseminated intravascular coagulation (DIC) with extensive bone marrow metastasis, resulting in death from cerebral hemorrhage. Although DCBM due to malignancy is rare with poor prognosis, it should be considered in malignancies associated with DIC, and prompt initiation of chemotherapy is the only way to improve the patient's prognosis.

## Graphical abstract

We present a case of disseminated carcinomatosis of the bone marrow from esophageal adenocarcinoma. The interosseous spaces of the bone marrow of the spinal vertebrae are occupied by poorly differentiated adenocarcinoma.

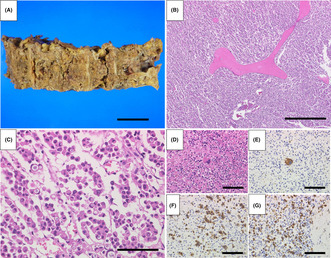

## 1 INTRODUCTION

Disseminated carcinomatosis of the bone marrow (DCBM) is a condition in which tumor cells from solid tumors extensively metastasize to the bone marrow, replacing the bone marrow tissue with tumor cells. Diffuse invasion of the bone marrow by tumor cells often results in disseminated intravascular coagulation (DIC) syndrome and microangiopathic hemolytic anemia. DCBM occurs in several tumors, including cancers of the stomach,^1^ breast,^2^ colon and rectum,^3^ prostate,^4^ tongue,^5^ head and neck,^6^ hepatocellular carcinoma,^7^ intrahepatic cholangiocarcinoma,^8^ duodenal,^9^ pancreatic,^10^ bladder,^11^ uterine cervical,^12^ and anal canal^13^ carcinomas. In a follow‐up study of 2235 curatively resected gastric adenocarcinomas, there were four cases (0.2%) with matching DCBM.^14^ However, the frequency of DCBM in malignant tumors other than gastric adenocarcinomas remains unclear.

The histological types of esophageal carcinoma are divided into squamous cell carcinoma and adenocarcinoma, the latter arising from Barrett's esophagus, which is glandular metaplasia of the esophagus. The incidence of esophageal adenocarcinoma is increasing worldwide; it accounts for more than 50% of all esophageal carcinomas in the United States and Europe and approximately 7% of cases in Japan.^15^ DCBM from esophageal carcinoma to the bone marrow is extremely rare and uncommon, not only in Japan but also worldwide.

In this report, we present the autopsy case of a patient with DIC syndrome and cerebral hemorrhage caused by DCBM from esophageal adenocarcinoma. To the best of our knowledge, this is the first of such cases.

## 2 CASE HISTORY

**FIGURE 1:**
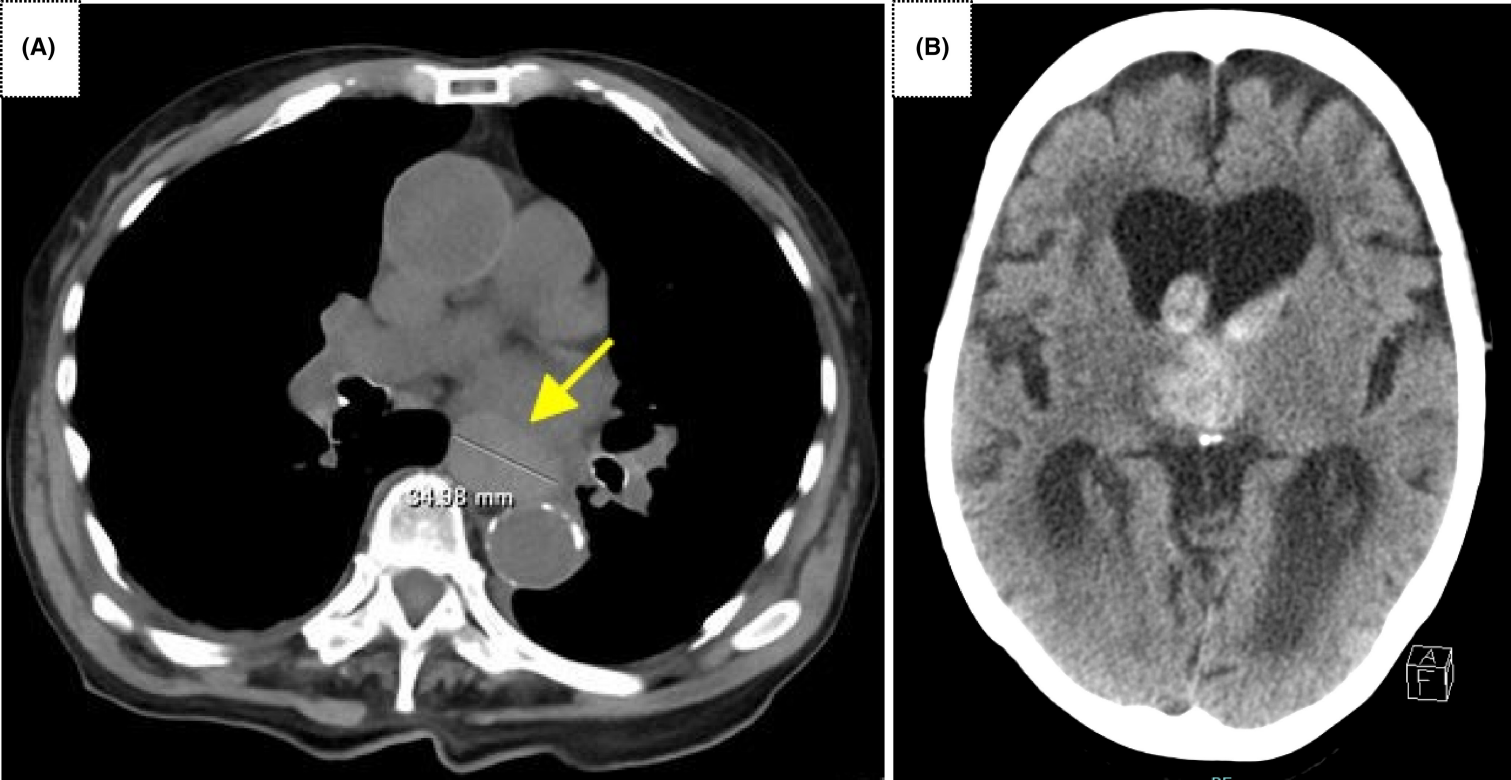
(A) Simple chest computed tomography (CT) scan showing a 35‐mm mass in the esophagus. Due to the presence of a hiatal hernia of the esophagus, the tumor was found to be more cephalad than its original location. (B) Simple head CT scan showing right thalamic hemorrhage and perforation of the lateral ventricle.

The patient was an 89‐year‐old woman under observation at another hospital for hypertension and osteoporosis. There was no family history of note. Her medical history and medication history included a prescription for a calcium channel blocker for hypertension, a bisphosphonate, and activated vitamin D for osteoporosis. Blood tests performed at the former hospital 2 months before admission revealed mild anemia with a hemoglobin level (Hb) of 11.3 g/dL and mild thrombocytopenia with a platelet (Plt) count of 19.0 × 10^4^/μL. She was referred to our hospital for easy fatigability, and an electrocardiogram at the time of admission showed atrial fibrillation. On admission, she had severe anemia with Hb levels of 3.6 g/dL and severe thrombocytopenia with a Plt count of 2.6 × 10^4^/μL. She also had a low fibrinogen level (55 mg/dL), an increased fibrinogen degradation product (FDP) level (225 μg/mL), and fragmented red blood cells in the peripheral blood, indicating DIC. Blood data at admission and thereafter are summarized in Table 1. Immature white blood cells were observed in the peripheral blood. Chest computed tomography (CT) performed on admission revealed a hiatal hernia of the esophagus and a mass in the middle esophagus (Figure 1A), suggestive of bone marrow metastasis of an esophageal tumor or a combination of an esophageal tumor and hematopoietic disease.

**TABLE 1:** Patient's laboratory data.

	On admission	Day 2	Day 5
WBC (/μL)	9400	9000	10,200
RBC (×10^4^/μL)	130	228	243
Hb (g/dL)	3.6	6.8	7.4
Ht (%)	12.5	19.8	21.3
PLT (×10^4^/μL)	2.6	1.3	0.9
PT (%)	64.6	63.1	60.2
PT (s)	14.9	15.1	15.5
PT INR	1.3	1.3	1.4
PT ratio	1.3	1.3	1.3
APTT (s)	25.6	33.2	34.5
Fib (mg/dL)	55	82	70
FDP (μg/mL)	225	298	324
d‐Dimer (μg/mL)	–	–	57.4
AT III	–	76.3	64.4
TP (g/dL)	5.6	5.3	5.0
Alb (g/dL)	3.1	3	2.6
T‐Bil (mg/dL)	0.9	1.9	2.6
AST (U/L)	147	167	203
ALT (U/L)	245	234	273
LDH (U/L)	570	785	1241
ALP (U/L)	48	50	68
BUN (mg/dL)	37.7	18.7	25.8
Cr (mg/dL)	1.0	0.8	0.7
CRP (mg/dL)	0.1	0.5	0.7

Abbreviations: Alb, albumin; ALP, alkaline phosphatase; ALT, alanine aminotransferase; APTT, activated partial thromboplastin time; AST, aspartate aminotransferase; AT III, anti‐thrombin III; BUN, blood urea nitrogen; Cr, creatinine; CRP, C‐reactive protein.; Fib, fibrinogen; Hb, hemoglobin; Ht, hematocrit; LDH, lactate dehydrogenase; PLT, platelet; PT, prothrombin time; RBC, red blood cell; T‐bil; total bilirubin; TP, total protein; WBC, white blood cell.

On admission, the patient was treated conservatively with blood transfusions for anemia and thrombocytopenia and fresh frozen plasma for DIC. Bone marrow aspiration was performed; however, an insufficient amount of bone marrow was obtained. Early in the morning on the fifth day of hospitalization, she suddenly lost consciousness; emergency head computed tomography (CT) revealed right thalamic hemorrhage and ventricular perforation (Figure 1B). Her state of consciousness worsened, and she died a few hours later. Upper gastrointestinal endoscopy and esophageal biopsy were scheduled but not performed. An autopsy was performed to confirm the histologic type of the esophageal tumor and bone marrow status.

**FIGURE 2:**
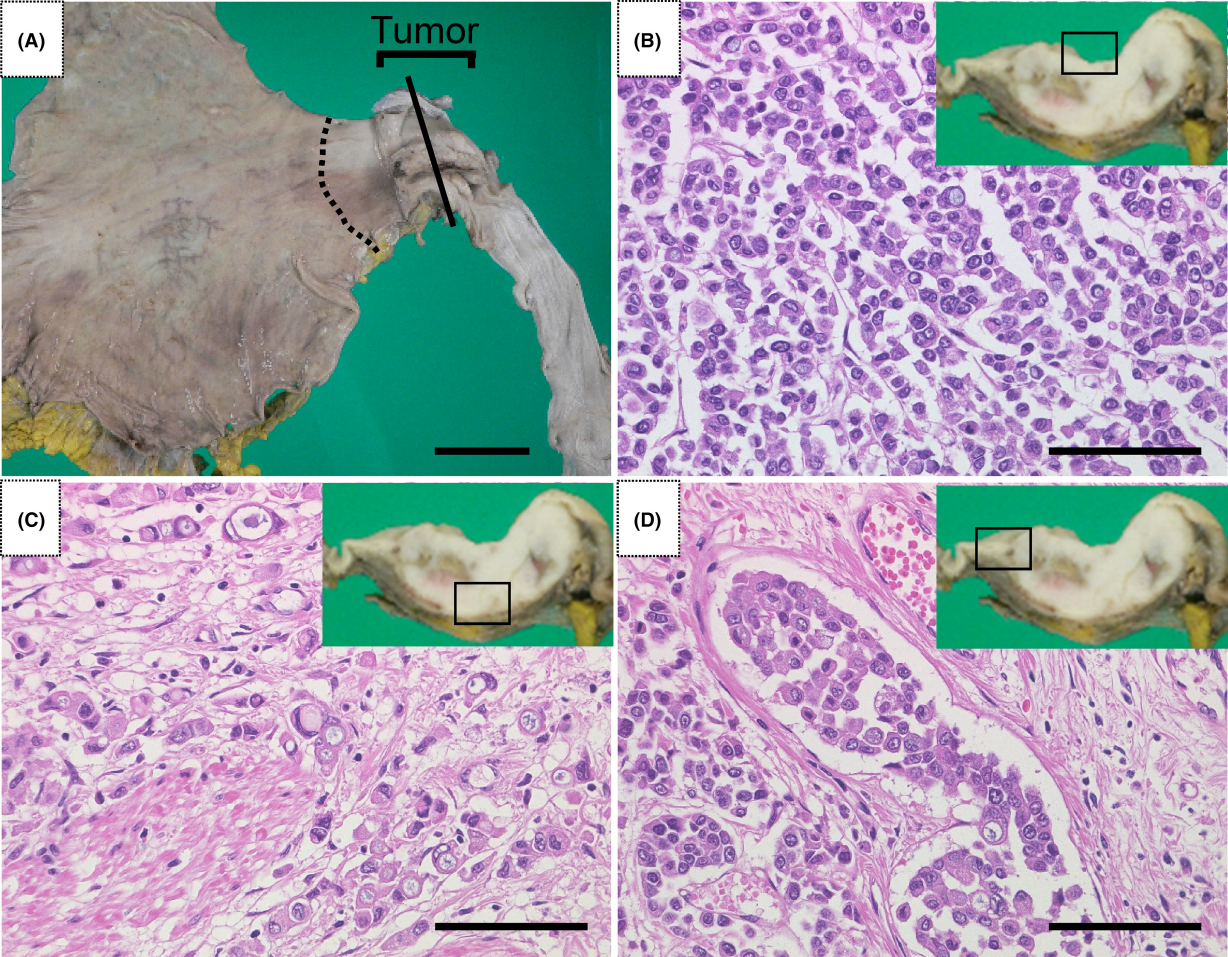
(A) Histological examination of specimens from the esophagus and stomach after formalin fixation. The dashed line indicates the histological gastroesophageal junction. A mass measuring approximately 5 × 3.5 cm and occupying three‐quarters of the circumference is seen at approximately 4 cm from the gastroesophageal junction to the mouth. The tumor was cut along the solid line, creating a cut surface of the tumor in insets (B), (C), and (D). (B) The tumor near the esophageal lumen side (rectangle in the inset) is mainly composed of poorly differentiated adenocarcinoma (×400). (C) The tumor in the esophageal muscularis propria (rectangle in the inset) is mainly composed of signet ring cell carcinoma (×400). (D) Tumor lymphovascular invasion is observed at the margins of the tumor (×400). Scale bars = 5 cm (A) and 20 μm (B, C, and D).

**FIGURE 3:**
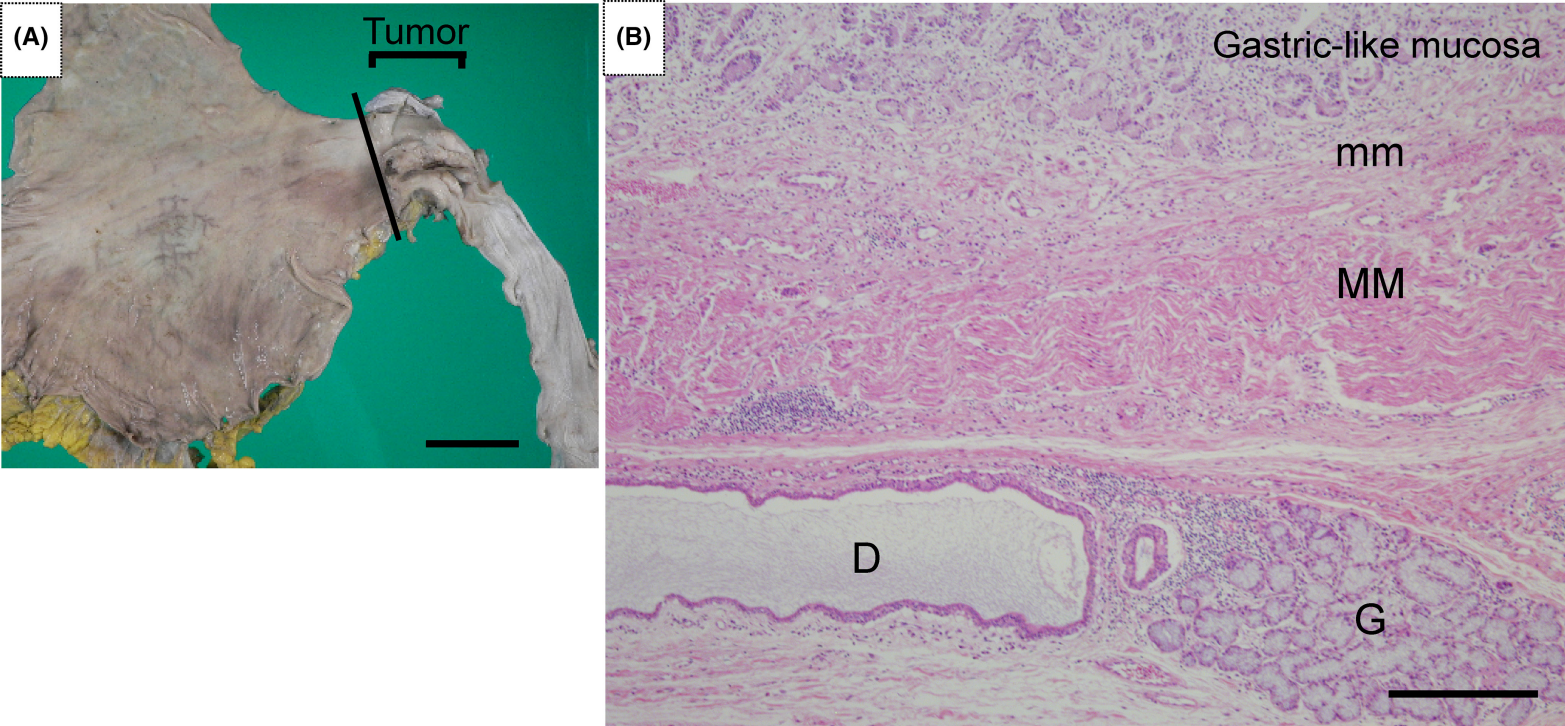
(A) Histological examination of specimens from the esophagus and stomach after formalin fixation. The esophagus was cut along the solid line, creating a cut surface on the most distal side of the tumor. (B) Low magnification of the most distal side of the tumor showing the coexistence of non‐neoplastic gastric‐like mucosal layers and esophageal submucosal glands, and duplication of the mucosal muscular layer (×40). mm and MM indicate superficial and deep mucosal muscular layer, respectively. D indicates the duct of the esophageal submucosal gland. G indicates the esophageal submucosal gland. Scale bars = 5 cm (A) and 100 μm (B).

Autopsy revealed a tumor (5 × 3.5 cm, 3/4 circumference, Siewert‐Stein classification type 1), which had been observed before death, in the lower esophagus 4 cm orally from the esophagogastric junction (Figure 2A). Although the tumor was located in the lower esophagus, it was considered a tumor in the middle esophagus on chest CT because of a hiatal esophageal hernia. Microscopically, the tumor was a poorly differentiated adenocarcinoma (Figure 2B) with some signet ring cell carcinomas (Figure 2C) and lymphovascular invasion (Figure 2D). Histological findings of the peritumoral tissue included squamous island‐like structures, coexistence of gastric‐like mucosal layers and esophageal submucosal glands, and duplication of the mucosal muscular layer (Figure 3), consistent with Barrett's esophagus (esophageal long axis, approximately 8 cm). Based on these findings, the tumor was diagnosed as esophageal adenocarcinoma in Barrett's esophagus. The adenocarcinoma had invaded the adventitia of the esophagus and metastasized to nearby lymph nodes.

**FIGURE 4:**
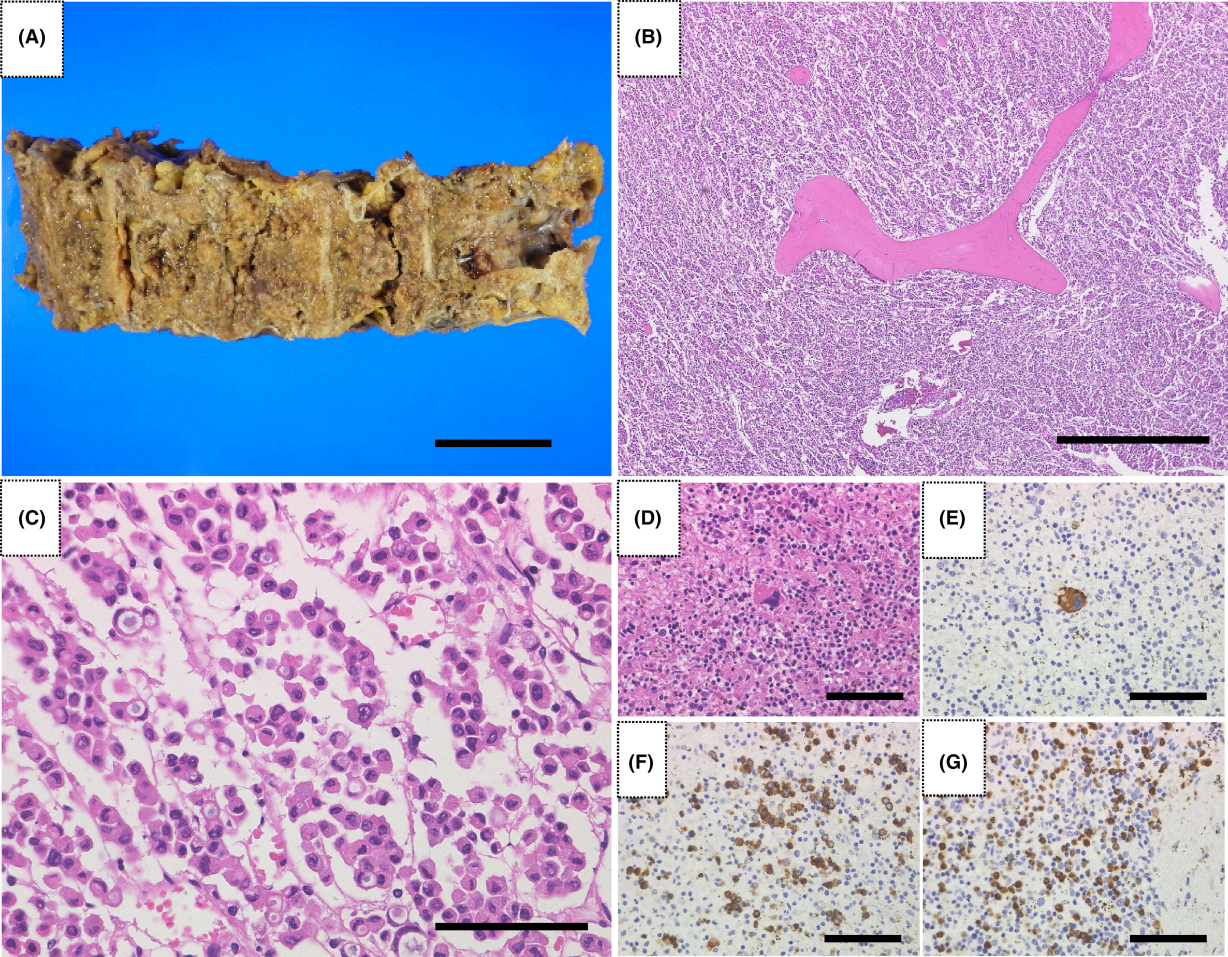
(A) In the cross section of the spinal vertebra, the marrow is white, suggesting hypercellular marrow. (B) Low magnification of the bone marrow of the spinal vertebrae showing no hematopoietic cells in the interosseous spaces, occupied by poorly differentiated adenocarcinoma (×40). (C) High magnification of poorly differentiated adenocarcinoma occupying the interosseous spaces of the spinal vertebrae (×400). (D) Hematoxylin–Eosin staining of the spleen showing megakaryocyte‐like giant cells (×400). (E) Immunohistological staining of the spleen with anti‐CD42b antibody showing megakaryocytes (×400). (F) Immunohistological staining of the spleen using anti‐CD71 antibody showing erythroblasts (×400). (G) Immunohistological staining of the spleen using anti‐MPO antibody showing granulocytes (×400). Scale bars = 5 cm (A), 200 μm (B), 20 μm (C), and 10 μm (D, E, F, and G).

The bone marrows of almost all vertebrae were grossly white (Figure 4A). Histologically, the poorly differentiated adenocarcinoma was diffusely invasive, and signet ring cell carcinoma was observed in some areas (Figure 4B,C). Normal hematopoietic tissue was significantly reduced in the bone marrow. Erythroblasts, immature granulocytes, and megakaryocytes were also observed in the liver and spleen, suggesting extramedullary hematopoiesis (Figure 4D–G). Based on these findings, the patient was diagnosed with DCBM. A 5‐mm metastatic lesion was found in the lower lobe of the right lung. No other metastases were found. There were no apparent findings in the heart, lungs, or aorta to explain the sudden death. Therefore, the right cerebral thalamic hemorrhage was considered the direct cause of death.

## 3 DISCUSSION

We reported an autopsy case of disseminated carcinomatosis of the bone marrow from an esophageal adenocarcinoma. In 1979, Hayashi et al.^16^ analyzed bone metastases from solid tumors and concluded that DCBM is a diffuse bone marrow metastasis of carcinomas. DCBM is characterized by anemia, DIC, juvenile granulocytes or erythroblasts in the peripheral blood, or angiopathic hemolytic anemia, and elevated lactate dehydrogenase and alkaline phosphatase levels. Gastric adenocarcinoma is the commonest cause of DCBM in Japan. Four of 2235 (0.2%) cases of surgically resected gastric adenocarcinomas in a single‐center study were cases of DCBM from gastric adenocarcinomas; all were poorly differentiated.^14^ The frequency of DCBM from cancers involving other organs except the stomach is unknown.^17^ A search on PubMed using “bone marrow carcinomatosis and DIC” as search words yielded 60 articles published over the past 40 years. Apart from gastric adenocarcinoma (35 cases), cases of DCBM from colorectal carcinoma (16 cases), pancreatic carcinoma (2 cases), prostatic carcinoma (6 cases), breast carcinoma (2 cases), gastroesophageal junction carcinoma (1 case), duodenal carcinoma (1 case), anal duct carcinoma (1 case), hepatocellular carcinoma (1 case), intrahepatic cholangiocarcinoma (1 case), and malignant pheochromocytoma (1 case) have been reported.

Approximately 26,000 cases of esophageal carcinoma are registered in Japan annually, of which esophageal adenocarcinoma accounts for 7%, corresponding to 1800 cases of esophageal adenocarcinoma per annum.^14^ Assuming that the frequency of DCBM from esophageal adenocarcinoma is equivalent to that from gastric adenocarcinoma (0.2%), approximately three to four cases of DCBM from esophageal adenocarcinoma should be registered annually. However, as there have been no case reports of DCBM from esophageal adenocarcinoma in Japan, DCBM from esophageal adenocarcinoma is less frequent than DCBM from gastric adenocarcinoma.

Given that esophageal adenocarcinoma arises from Barrett's mucosa, in which the squamous epithelium of the esophagus is converted into a columnar epithelium extending from the stomach into the esophagus in a continuous fashion, there is a high histological similarity between gastric and esophageal adenocarcinomas. However, despite the histological similarities between gastric adenocarcinoma and Barrett's esophageal adenocarcinoma, various studies have shown that the mechanisms of tumorigenesis differ.^18^, ^19^ Differences in the nature of adenocarcinoma tumor cells may result in differences in the frequency of DCBM from gastric and esophageal adenocarcinomas.

Although not listed in PubMed, only one case of esophageal adenocarcinoma with DCBM has been reported.^20^ The patient was a 73‐year‐old man with DIC caused by DCBM from a poorly differentiated primary adenocarcinoma of the esophagus. His laboratory data at admission were as follows: Hb, 7.7 g/dL; Plt, 2.0 × 10^4^/μL; albumin, 3.2 g/dL; prothrombin time, 19.5 s; activated partial thromboplastin time, 43 s; international normalized ratio, 1.68; fibrinogen, 130 mg/dL; and d‐Dimer, 2.9 μg/mL. The patient received intensive supportive care for DIC, followed by first‐line chemotherapy with 5‐fluorouracil, leucovorin, and oxaliplatin (FOLFOX). Treatment of the underlying malignancy resulted in stabilization of the DIC, followed by patient discharge. Recently, the successful treatment of DCBM from various malignancies has been reported. In the past 3 years, two English‐language papers have reported that anticoagulation and chemotherapy for DCBM from gastrointestinal adenocarcinoma have led to the discharge of patients from the hospitals.^21^, ^22^

Disseminated carcinomatosis of the bone marrow is associated with a high rate of DIC, and its prognosis is very poor. Aggressive treatment of malignancy improves DCBM prognosis.^20^, ^23^ In DCBM, chemotherapy should be administered promptly before the patient's general condition deteriorates, as rapid deterioration of the patient's general condition may make it difficult to initiate chemotherapy. A bone marrow biopsy is commonly performed before initiating treatment for DCBM. However, it has been reported that PET‐CT outperforms bone marrow biopsy in terms of sensitivity and specificity in detecting cancer cell invasion into the bone marrow.^17^, ^24^ PET‐CT is also useful for estimating the primary organ in patients with DCBM and unknown primary cancer and may improve DCBM prognosis by providing a first screening method for bone marrow invasion of cancer and prompting the initiation of treatment for DCBM.

In general, DIC associated with malignant tumors is considered to be hyperfibrinolytic DIC, which is caused by the activation of extrinsic coagulation by tissue factors derived from tumor cells. Acute promyelocytic leukemia (APL) is well known as a typical disease‐causing hyperfibrinolytic DIC, and severe DIC in APL can cause cerebral hemorrhage. In the case of DIC associated with DCBM presented here, FDP was high, fibrinogen was low, and the platelet count decreased to about 10,000/μL, suggesting that the patient was prone to cerebral hemorrhage due to hyperfibrinolytic DIC. In addition, the patient had a history of hypertension, and there was a possibility of atherosclerosis in the cerebral arteries. Furthermore, an electrocardiogram at the time of admission showed atrial fibrillation, so the possibility that a hemorrhagic cerebral infarction occurred due to an embolization of a minute thrombus in the cerebral artery cannot be ruled out. However, since the brain was excluded from the autopsy, no histological search was performed, and the details are unknown.

DCBM is extremely rare and uncommon, not only in Japan but also worldwide. Therefore, this case report will provide clinicians with further knowledge about this condition. Moreover, this report proves that although DCBM is very rare, it can occur in esophageal adenocarcinomas and adenocarcinomas of other organs.

## AUTHOR CONTRIBUTIONS

**Daisuke Suzuki:** Formal analysis; writing – original draft. **Shiori Meguro:** Formal analysis; writing – original draft. **Shin Furusawa:** Data curation; formal analysis. **Nanako Hashimoto:** Formal analysis. **Hideya Kawasaki:** Formal analysis. **Isao Kosugi:** Formal analysis. **Yasunori Enomoto:** Writing – review and editing. **Mayu Fujihiro:** Writing – review and editing. **Hiroe Tsukui:** Writing – review and editing. **Toshihide Iwashita:** Formal analysis; writing – original draft.

## FUNDING INFORMATION

This work was supported by the Grants‐in‐Aid for Scientific Research C (grant numbers 20K07370 and 21K06947) from the Japan Society for the Promotion of Science. The funder provided financial support for the study but was not involved in the study design; data collection, analysis, and interpretation; or writing of the manuscript.

## CONFLICT OF INTEREST STATEMENT

The authors declare no conflicts of interest regarding the publication of this article.

## ETHICS STATEMENT

This case report was approved by the Ethics Committee of the Chutouen General Hospital (approval number: 1226230316).

## CONSENT

We obtained written informed consent to publish the case details from the patient's family.

## Data Availability

The authors declare that all the relevant data were included in this report and are available herein.
